# Determinants of immunisation in children with sickle cell disease in Libreville

**DOI:** 10.4102/jphia.v16i1.663

**Published:** 2025-02-13

**Authors:** Edgard B. Ngoungou, Ulrick J. Bisvigou, Jean Engohang-Ndong, Valessa Anguezomo, Maghendji N. Sydney, Euloge Ibinga

**Affiliations:** 1 Department of Community and Social Medicine, Faculty of Medicine, University of Health Sciences, Owendo, Gabon; 2 Research Unit in Epidemiology of Chronic Diseases and Environmental Health (UREMCSE), Faculty of Medicine, University of Health Sciences, Owendo, Gabon; 3 Department of Biology, Kent State University, New Philadelphia, United States of America

**Keywords:** attitude, Gabon, knowledge, sickle cell disease, vaccination coverage

## Abstract

**Background:**

Infectious diseases are frequent and sometimes deadly in sickle cell disease (SCD) patients. Some of these infectious diseases could be avoided through immunisation, but an immunisation schedule for children with SCD is not available in Gabon.

**Aim:**

This study looked into the determinants of immunisation in children with SCD in Libreville.

**Setting:**

This work was performed in five healthcare facilities in Libreville.

**Methods:**

A cross-sectional study on knowledge, attitudes, and practices was conducted from February 2019 to September 2019 in Libreville healthcare facilities, targeting children under 18 years with SCD.

**Results:**

A total of 172 parents of children with SCD participated. The average age of children was 7.1 ± 4.2 years, with a sex ratio of 1:36. Immunisation status was considered complete for 87.9% (95% CI = 79.8–93.1) according to the Expanded Programme of Immunisation (EPI) schedule. Only 49 (28.5%) parents understood SCD complications, and 39 (22.7%) knew how to prevent them. Immunisation coverage was better for children near public health centres (*p* = 0.008). For non-EPI vaccines, coverage improved for children of married parents (*p* = 0.041) and those seen by paediatricians in private facilities (*p* = 0.046). Multivariate analysis indicated that marital status, a lack of knowledge, facility access, and high vaccine costs predicted immunisation coverage.

**Conclusion:**

Immunisation coverage of children with SCD was better than the national immunisation coverage in Gabon.

**Contribution:**

This study unravels the need for Gabon to improve its immunisation programmes in public healthcare facilities.

## Introduction

Sickle cell disease (SCD) or sickle cell anaemia (SCA) is an autosomal disorder characterised by the presence of abnormal haemoglobin in red blood cells. Sickel cell disease is one of the most common severe inherited disorders affecting millions of people worldwide. According to the World Health Organization (WHO), 4.5% of the world population is carrier of the sickle cell gene. Every year, more than 300 000 children are born with the severe form of this disease, and it is estimated that this number could reach 400 000 in 2050.^1^ By far, Africa and India are the regions most affected by this disease. Approximately, 305 800 babies were born with SCD in 2010, of which two-thirds were born in Africa. It is thought that this number could increase by 25% to approximately 404 200 by 2050.^2^ The prevalence of SCD is 5% – 10% in Burkina Faso and 0.6% in Cameroon.^3,4^ In Gabon, this congenital health condition affects 2% of the population, which represents approximately 2000 newborns per year^5^; the sickle cell trait is approximately 21,1% of subjects over the age of 15 years are sickle cell trait carriers^6^ while this number is 1.8% in newborns who were screened for the disease.^7^

Sickle cell disease is associated with high level of mortality and morbidity, mainly in children under 5 years old.^2^ Morbidity and mortality because of infectious diseases in children with SCD are very high in Africa. This is illustrated by the fact that bacterial infections are responsible for 20% – 50% of mortality in children with SCD under 5 years old.^8^ Furthermore, bacterial infections are shown to be the cause of 21% of hospitalisations and 7.5% of deaths in Burkina Faso.^3^ Similarly, bacterial infections are responsible for as high as 36% of hospitalisations and 4.8% of mortality in children with SCD in Democratic Republic of Congo (DRC).^9^ Sickle cell disease accounts for 7.2% of deaths in paediatric hospital services in Gabon, with a lethality rate of 3.6%; the main causes of death are anaemia, infections and complications related to transfusion.^10^ When looking into data to determine the most commonly affected body organs in SCD infected patients, it appears that lungs were the most affected parts along with the urinary tract and the bones.^11^ It is now well established that anaemia, susceptibility to infections, acute chest syndrome that results from a vaso-occlusive crisis, acute pain and organ failure are associated with complications of SCD and are responsible for morbidity and mortality of the disease. In Côte d’Ivoire (Ivory Coast), a study found that the most common infections in SCD patients are those that are caused by infectious agents for which a vaccine is available including *pneumococcus, haemophilus, meningococcus, hepatitis* B virus, and *salmonella.*^12^ This statement also holds true for normal patients without SCD. The same study showed that respiratory infections occurred in 61% of cases analysed. In Gabon, the carriage of *Streptococcus* pneumoniae, *Staphylococcus* aureus and *Haemophilus influenzae* was similar in children with SCD or normal children^13^; but pulmonary and urinary infections are responsible for 3.6% of mortality in children with SCD. However, only few studies have shown that routine immunisation of children against *Pneumococcus* can decrease the risks of pulmonary infections from pneumococcal bacteria of about 70% in SCD children under five.^14,15^ In many countries around the world and in countries where SCD is endemic, vaccination recommendations specific to children with SCD are not always clear. World Health Organization recommends a comprehensive management of SCD through education of parents, adequate nutrition and hydration, use of prophylactic antibiotics and antimalarial drugs, use of folic acid supplementation, administration of specific vaccines, recurrent medical visits and medical monitoring, and early screening and management of complications.^16^ In spite of the fact that causes of death among children with SCD are poorly documented in Africa, it is established that the role of bacterial sepsis is significant.^17,18^ Thus, in order to prevent frequent infections in patients with SCA, it seems important to understand potential associated factors such as economic and/or social conditions of patients and their families as such factors could constitute barriers or accelerators to the effectiveness of immunisation schedule in SCD children. In Gabon, because of the lack of specific national policies and recommendations to paediatricians, there is no database or even simply data about immunisation coverage, regarding Expanded Programme of Immunisation (EPI) schedule or other recommended vaccines. The country does not have any national recommendation about immunisation for SCD children. There is also no data on knowledge, attitude and perceptions of parents’ with SCD children about vaccination and other means to avoid complications from this disease or factors that could influence the administration of vaccines in SCD children. There is no national recommendation for children with SCD. The EPI schedule is for any children. There is no database to compare the immunisation coverage of children with SCD to that of children without SCD.

Sickle cell disease affects the spleen, which is an accessory organ of the immune system. As a result, people with SCD have weakened immune systems and are more likely to get sick. Getting vaccinated, avoiding contact with sick people, and washing your hands frequently are good ways to stay healthy.^19^

Expanded Programme of Immunisation is an initiative of the WHO and United Nations Children’s Fund (UNICEF), which was established in 1974 to increase the uptake of routine childhood vaccines worldwide. In 1977, the goal was set to make immunisation against diphtheria, pertussis, tetanus, poliomyelitis, measles and tuberculosis available to every child in the world by 1990. As a consequence of the implementation of EPI worldwide, there has been a major reduction in deaths and hospital admissions from measles and neonatal tetanus in developing countries. The success of the programme represents a major public health achievement. However, many challenges are still real and need to be addressed.^20^

In Gabon, the EPI was launched in Libreville in 1978. It was under the responsibility of the Service des Grandes Endémies (SGE), with its staff and its vaccination teams until 1986. This programme was gradually extended to the whole country.^21^ Interestingly, since 1986, the vaccination programme has been moved under the responsibility of health facilities, leaving EPI with the responsibility to coordinate immunisation activities via the General Director of Health, Ministry of Health, and thus integrated into the health system.^22,23^ Thus, under that model, the programme was fully implemented in 1990.

The Gabon EPI schedule includes the Bacillus Calmette-Guerin (BCG) to prevent tuberculosis, the oral polio vaccine (OPV) that immunises children against poliomyelitis, the pentavalent-vaccine (including antigens against diphtheria, tetanus, pertussis, hepatitis B, and *Haemophilus influenzae* type b), the measles and yellow fever vaccines for children by the time they reach 9 months of age (Table 1). The EPI schedule is composed of 10 appointments, which are distributed starting at birth through roughly the 12th month of a child. Gabon is not part of the Global Alliance for Vaccines and Immunisation because of the country being listed as an upper-median income country. Therefore, Gabon has to fund and implement its own EPI programme throughout the country. Immunisation is free of charge for children less than 1-year-old in Gabon. However, parents have to pay out of pocket for all other vaccines that are recommended by paediatricians, including but not limited to Pneumococcal polysaccharide vaccine (PPSV23), anti-Rotavirus, anti-Typhoid, Meningo a + c, Penta booster, and Hep B booster.

**TABLE 1:** Vaccination schedule for Gabon’s Expanded Programme of Immunisation.

Child’s age	Vaccines required	Targeted diseases
Birth	BCG, OPV0	Tuberculosis, poliomyelitis
6 weeks	OPV1, DTP-Hib-Hep B1	Poliomyelitis, diphtheria, tetanus, pertussis, hepatitis B, *Haemophiles influenzae* meningitis
10 weeks	OPV2, DTP-Hib-Hep B2	Poliomyelitis, diphtheria, tetanus, pertussis, hepatitis B, *Haemophiles influenzae* meningitis
14 weeks	OPV3, DTP-Hib-Hep B3 and IPV	Poliomyelitis, diphtheria, tetanus, pertussis, Hepatitis B, *Haemophiles influenzae* meningitis, polio
9 months	M, YF	Measles and yellow fever

BCG, Bacillus Calmette-Guerin; OPV, oral polio vaccine; DTP, Diphtheria-Tetanus-Pertussis; IPV, inactivated polio vaccine.

The national EPI services are readily available in public health facilities as these are government-owned facilities. Occasionally, the national EPI services are offered in a few private clinics located in Libreville, the capital of Gabon. Nevertheless, none of the health facilities, public or private, are really equipped with a structured data capture in this programme to save the data of patients and retrieve them as needed.

The aim of our study was to assess the immunisation coverage of children with SCD using the EPI schedule and other vaccines and to unravel the potential determinants of immunisation in children with SCD in Libreville, Gabon.

## Research methods and design

### Study period

The study was conducted from 25 February 2019 to 26 August 2019.

### Study design

This study employed a descriptive cross-sectional design focusing on knowledge, attitudes, and practices. The decision to adopt this approach stemmed from the gaps and insufficient data regarding the exact number of vaccinations, knowledge of follow-up procedures, vaccination or immunisation schedules, and recommended immunisations for children with SCD. Therefore, the objective of this study was to utilise a concise, yet intensive, 6-month period for data collection to estimate and bridge the knowledge gap concerning the vaccination coverage of children with SCD who regularly visit public and private health facilities for the Extended Immunisation Programme and/or other vaccines in Libreville, Gabon. To achieve this, we concentrated our study on healthcare facilities that provided paediatric services and where patients with SCD frequently received medical consultations and/or hospitalisation.

### Study population

The participants of this study included all children under the age of 15 years who were admitted to or consulted at selected hospitals in Libreville during the study period, regardless of the reason. In the absence of specialised care centres, children with SCD are monitored in all health facilities. Therefore, university hospitals were chosen for the study. More specifically, the study was offered to children seen during paediatric consultations or those hospitalised in one of the selected paediatric departments. Also, the study included only children whose parents or legal guardians provided consent, specifically, children with SCD under the age of 18 years.

### Study setting

The research was conducted at five healthcare facilities, in Libreville, the administrative capital of Gabon. About one-third of the population of Gabon lives in Libreville. This city houses the reference hospitals of the country, including university hospitals (CHU), military hospitals, and polyclinics. The paediatric services of Centre Hospitalier Universitaire de Libreville (CHUL-Pédiatrie), the Clinique BIE-ONDO, the paediatrics unit of the Hôpital d’Instruction des Armées Omar Bongo Ondimba (HIAOBO-Pédiatrie), the paediatrics unit of Centre Hospitalier Universitaire Mère-enfant Fondation Jeanne Ebori (CHUME-Pédiatrie), the Emergency unit of Centre Hospitalier Universitaire d’Owendo (CHUO-Urgences). These healthcare facilities were all selected for their unique ability to provide expertise in the treatment of sickle cell patients.

### Inclusion criteria

Children with SCD, regardless of the reason, for consultation or hospitalisation, aged at least 6 months and less than 18 years old, who were present in the facility at the time of this study were included as part of the target population.

### Exclusion criteria

This study excluded all children under 6 months and over 18 years of age. The main exclusion criteria comprised children diagnosed with thalassaemia or glucose-6-phosphate dehydrogenase deficiency, as well as refusals or absence from parents or tutors to participate in the study.

### Target population

This study included children with SCD who were under 18 years old but at least 6 months old. The target population comprised children with SCD who were either hospitalised patients or patients who came into the healthcare facility for an unscheduled medical consultation, a scheduled medical visit, or called in by their paediatrician. Parents were systematically provided with an informed consent form during the medical consultation or during the hospitalisation. Only patients whose parents or legal guardians signed the informed consent form were given a questionnaire afterwards. During the hospital visit and hospital stay, as children were examined, parents were asked questions about the prevention of SCD crisis, their demographic information, and their access to healthcare facilities and immunisation.

### Method of investigation

During this study, we used a tested questionnaire (tested in the Université des Sciences de la Santé, Gabon) that was made up of 40 questions. The questionnaire was developed by the Epidemiology Research Unit ‘Unité de Recherche en Epidémiologie des Maladies Chroniques et Santé Environnement (URMCSE)’ of the ‘Université des Sciences de la Santé’ in Gabon. After obtaining consent from each of the participants, interviews were conducted. Each of the interviews carried out during this study lasted less than 20 minutes. During the interviews, parents or legal guardians were asked questions about their socio-demographic background, SCD child immunisation history, and SCD child medical history, including circumstances of discovery of infectious diseases, infection types, frequency of occurrence of infectious diseases, and surgery history of the child. Furthermore, parents or legal guardians were asked about their awareness, access, and accessibility to vaccination. More specifically, they were asked about their knowledge and attitude with regard to SCD acute manifestations and chronic complications, the ways to avoid these complications, EPI vaccine, and preventable diseases. They were also asked about the rumours they had heard regarding vaccination, access, and accessibility to vaccination. Information gathered during these interviews of patients and parents or legal guardians of SCD children allowed us to build a database on the sought questions.

For this study, children and parents were met once and only immunisation records found in the health booklet of patients were taken into account. The information collected was reported in a standardised data collection sheet and saved in a database. The socioeconomic status of patients was sorted by family income, with low-income families making less than $180.00/month, median-income families with revenues ranging between $180.00 and $541.00/month, and high-income families making over $541.00/month.

Children who had received all vaccines recommended by the Gabonese EPI schedule by the age of 12 months and had received the associated boost shots according to their age were considered up-to-date and well-vaccinated.

### Study sample size

This study included dyads of parents and children, using a non-probabilistic sampling. The sample size was calculated according to the objectives. The minimal sample size (required sample size) was calculated using Schwartz formula:

$$N={\left(Z\alpha /\Delta \right)}^{2}.[p(1-p)]$$ [Eqn 1]

Where *Z*α value is based on 95% confidence interval = 1.96. Δ = precision = 0.05. *p* = proportion of children with SCD completely immunised with EPI schedule = 0.1 (our hypothesis).

In our study, the minimal sample size was 138 dyads.

### Statistical analysis

The data collected were saved in a database created with EPI data software version 4.6.0.0. Analyses were performed using *R* Statistical Software (version 3.6.2; *R* Core Team 2020). Quantitative variables were described using the average and standard deviation, while qualitative variables were represented as percentages and confidence intervals. The average was compared using the Student’s *t*-test and frequencies were analysed with the Chi-square-test (χ^2^-test). A multivariate analysis was conducted using a step-down logistic regression model, with a significance level of 0.05.

### Ethical considerations

The study obtained ethical clearance from the University of Health Sciences of Libreville and authorisation from the Gabonese Ministry of Health. All the patients and/or parents and/or caregiver(s) were provided with written informed consent and/or assent (as applicable) at enrolment. If a parent did not agree to participate in this study, the hospital visit of their child simply continued to follow the standard course of action for a typical hospital visit without any further interruption or ask. Privacy and confidentiality were maintained throughout the duration of this study and after.

Personal information was not communicated and was not taken into account in the data analysis. There was no risk to individuals from participating in this study.

## Results

A total of 172 subjects consisting of dyads of parent and child were included in the study.

### Socio-demographic and clinic status of sickle cell disease children

The socio-demographic characteristics of children with SCD and vaccine status are summarised in Table 2. Immunisation records were collected from 172 parent-child couples with SCD who were routinely seen by paediatricians in five healthcare facilities in Libreville. Of the 172 total patients, 135 (78.5%) were seen in government-owned health centres, while 37 (21.5%) were seen in private health centres. The mean age of children was 7.1 ± 4.2 years. Almost 57.6% (*n* = 99) were boys and the sex ratio (male : female) was 1:4. Parents of SCD patients were mostly living in domestic partnerships. Indeed, while 47 (27.3%) children were living with married parents, 60 (34.9%) were living in the same house as their unmarried parents, and 65 (36.8%) lived with single parents. Based on socioeconomic status, 89 out of 172 (51.8%) were low-income families, while 80 out of 172 (46.5%) had median income. Only 3 (1.7%) families surveyed fell in the high-income socioeconomic group.

**TABLE 2:** Socio-demographic characteristics, medical history and vaccine coverage of children with sickle cell disease (*N* = 172).

Variables	Frequency (*n*)	%	CI_95%_
**Children’s gender**			
Male	99	57.6	50.0–65.0
Female	73	42.4	35.0–50.0
**Age (years)**			
0–5	65	38.0	31.0–46.0
5–10	64	37.0	30.0–45.0
10–16	43	25.0	19.0–32.0
**Type of facilities**			
Public health centres	135	78.5	71.0–84.0
Private clinic	37	21.5	16.0–29.0
**Legal guardian**			
Both parents	82	48.0	40.0–55.0
Mother	73	42.0	35.0–50.0
Grandmother	8	4.7	2.2–9.3
Father	8	4.7	2.2–9.3
Grandfather	1	0.6	0.0–3.7
**Socio-economic level**			
Low	89	51.8	44.0–59.0
Medium	80	46.5	39.0–54.0
High	3	1.7	0.5–5.4
**Nationality**			
Gabonese	164	95.3	91.0–98.0
Other	8	4.7	2.2–9.3
**Chronic complication because of SCD**			
No	150	87.2	81.0–92.0
Yes	22	12.8	8.4–19.0
**Health or vaccine card**			
Yes	107	62.0	54.0–69.0
No	65	38.0	31.0–46.0
**Immunisation status according to EPI schedule**			
Complete	95	89.0	81.0–94.0
Not complete	12	11.0	6.2–19.0
**Vaccination out of EPI schedule**			
Yes	75	70.0	60.0–78.0
No	32	30.0	22.0–40.0
**Vaccination received out of EPI schedule**			
PPSV23	20	27.0	17.0–38.0
Anti-Rotavirus	29	39.0	28.0–51.0
Anti-Typhoid	30	40.0	29.0–52.0
Meningo a + c	34	45.0	34.0–57.0
Penta booster	47	63.0	51.0–73.0
Hep B booster	40	57.0	45.0–69.0

SCD, sickle cell disease; EPI, Expanded Programme of Immunisation; PPSV, Pneumococcal polysaccharide vaccine; CI, confidence interval.

Out of 172 children with SCD, 107 had their immunisation records correctly documented on their health booklet. A closer look at that number revealed that out of the 107 children with proper immunisation records, 94 (87.9%) of them had their vaccines up-to-date and in alignment with EPI standard (95% confidence interval [CI] = 79.8–93.1).

#### Parent knowledge about sickle cell disease and complications

Knowledge of parents about SCD was obtained during their interview. Our findings are summarised and displayed in Table 3. Overall, our study shows that a limited number of parents claimed to know something about SCD complications. More specifically, only 49 (28.5%) out of 172 families interviewed asserted to have knowledge of SCD complications. Similarly, we found that only 39 parents out of 172 reported to know how to prevent SCD complications (22.7%). In contrast, 133 (77.3%) parents declared to not know how to deal with SCD complications.

**TABLE 3:** Knowledge of parents about sickle cell disease complications, vaccine and immunisation session (*N* = 172).

Questions	Frequency (*n*)	%	CI_95%_
**Do you know SCD manifestations?**			
No	63	36.6	29.4–43.8
Yes	54	31.4	24.5–38.3
Partially	55	32.0	25.0–38.9
**Are you aware of SCD complications?**			
No	123	71.5	64.8–78.3
Yes	49	28.5	21.7–35.2
**Do you know how to prevent complications?**			
No	133	77.3	71.1–83.6
Yes	39	22.7	16.4–28.9
**Do you know SCD severity?**			
No	15	8.7	04.5–12.9
Yes	150	87.2	82.2–92.2
Partially	7	4.1	01.1–07.0
**Do you know how to prevent crisis?**			
No	72	41.9	34.5–49.2
Yes	91	52.9	45.4–60.4
Partially	9	5.2	01.9–08.6
**Have you ever heard about vaccine?**			
Never	1	0.6	00.0–02.8
Vaguely	3	1.7	00.4–04.7
Yes	168	97.7	95.4–99.9
**Do you know what a vaccine is?**			
No	33	19.2	13.3–25.1
Not really	14	8.1	04.1–12.2
Yes	125	72.7	66.0–79.3
**Is it important to be vaccinated?**			
No	4	2.3	00.1–04.6
Not really	3	1.7	00.4–04.7
Yes	165	96.0	93.0–98.9
**What is the age for getting immunisation?**			
Adolescent	40	23.3	19.9–29.6
Adult	105	61.0	53.8–68.3
Newborn	1	0.6	00.0–02.8
Infant	26	15.1	09.8–20.5
**Do you know the meaning of EPI?**			
No	119	69.2	00.0–03.7
Not really	17	9.9	05.4–14.3
Yes	36	20.9	62.3–76.1
**Do you know the EPI schedule?**			
No	20	11.6	06.8–16.4
Partially	18	10.5	05.9–15.0
Yes	134	77.9	71.7–84.1
**What is your main source of knowledge?**			
School	2	1.2	00.2–03.8
Reading the health book	8	4.7	02.2–08.6
Media	10	5.8	03.0–10.1
Family discussion (word of mouth)	27	15.6	10.8–21.7
Health workers	125	72.7	65.6–78.9
**Why vaccinate with other vaccine out of EPI?**			
Be healthy	137	79.7	73.1–85.2
Don’t know	21	12.2	07.9–17.8
Trip aboard	14	8.1	04.7–12.9
**What is the reason for immunisation refusal?**			
Others	20	11.6	07.5–17.0
High cost	118	68.6	61.7–75.5
Distance to healthcare facility	9	5.2	14.9–27.0
Mistrust in the vaccine	2	1.2	01.9–08.6
Don’t know	23	13.4	08.3–18.5
**What was the most annoying thing during immunisation session?**			
Bad reception	12	7.0	03.8–12.0
Information access	23	13.4	08.8–25.0
Distance to vaccination centres	31	18.0	13.0–25.0
Very long waiting time	89	52.3	44.0–59.0
Without opinion/Not pronounced	17	9.9	6.0–16.0

SCD, sickle cell disease; EPI, Expanded Programme of Immunisation; CI, confidence interval.

#### Knowledge of parents about immunisation and vaccination

The knowledge of parents about immunisation is compiled and displayed in Table 3. Most parents (97.7%) had heard about vaccines, but only 20.9% knew the meaning of EPI. A total of 129 (75%) of parents had heard rumours concerning immunisation or vaccines, and 69.8% of them did not agree with these rumours (Table 4).

**TABLE 4:** Knowledge of parents about rumours related to vaccination (*N* = 129) in Libreville, Gabon.

Questions	Frequency (*n*)	%	CI_95%_
**Type of rumour**			
Experimental vaccine	5	3.9	1.4–8.4
Expired vaccine	15	11.6	06.8–16.4
Cause illness	90	69.8	62.9–76.6
Useless	5	3.9	01.0–06.8
Kills	6	4.6	01.5–07.8
Others	8	6.2	01.5–07.8
**Agree with rumour**			
No	90	69.8	62.9–76.6
Yes	18	13.9	08.8–19.1
Not really	21	16.3	10.8–21.8
**Justified rumour**			
No	88	68.2	61.3–75.2
Yes	19	14.7	09.4–20.0
Not really	22	17.1	14.9–27.0
**Credible rumour**			
No	94	72.9	66.2–79.5
Yes	17	13.2	08.1–18.2
Not really	18	13.9	08.8–19.1

CI, confidence interval.

#### Socio-demographic data and immunisation status

There was no association between the type of healthcare facility and the immunisation status of the children (*p* = 0.105). The socioeconomic status of families was not associated with either the EPI coverage (*p* = 0.862), the Penta booster (*p* = 0.815), or the PPSV23 coverage (*p* = 0.259).

Similarly, there was no association between the marital status of parents and the immunisation status of children (*p* = 0.925) based on the EPI schedule. However, we observed a significant association between marital status and PPSV23 immunisation status. Indeed, children living with married parents (46.4%) had better coverage for PPSV23 compared to children living with single parents (20.8%) or living with parents in a domestic partnership (17.4%), *p* = 0.041. Furthermore, we found an association between the Penta reminder and the marital status of parents. Thus, children living with married parents had a better Penta recall coverage (46.8%) compared to children living with single parents (26.6%) or living with parents in a domestic partnership (26.7%), with *p* = 0.027.

There was a clear association between socioeconomic status of parents and the frequency of complications. Hence, we observed that complications were more frequent among children coming from median-income families (72.0%) than in those coming from low-income families (27.3%), *p* = 0.028, while SCD children coming from high-income group presented no complications. Patients coming from low-income families were a bit younger than children from the median-income group, with an average age of 6.5 ± 4.3 years versus 7.0 ± 4.3 years, respectively (Table 5).

**TABLE 5:** Comparison of full vaccination within and outside the Expanded Programme of Immunisation schedule, along with other variables, in children with sickle cell disease residing in Libreville.

Characteristics	Socio-economic level				*P* *
	Low (*n* = 89)†		Medium-high (*n* = 83)†		
	*n*	%	*n*	%	
**Sanitary facilities**	-	-	-	-	0.008
Public health centres	77	87.0	58	70.0	-
Private clinic	12	13.0	25	30.0	-
**Legal guardian**	-	-	-	-	0.018
Both parents	35	39.3	47	56.6	-
Mother	48	53.9	25	30.1	-
Grandmother	3	3.4	5	6.0	-
Father	3	3.4	5	6.0	-
Grandfather	0	0.0	1	1.2	-
**Marital status of parents**	-	-	-	-	0.049
Single	41	46.0	24	29.0	-
Concubinage	29	33.0	31	37.0	-
Married	19	21.0	28	34.0	-
**Nationality**	-	-	-	-	0.007
Gabonese	81	91.0	83	100.0	-
Others	8	9.0	0	0.0	-
**Compilation of SCD**	-	-	-	-	0.014
No	83	93.3	67	81.0	-
Yes	6	6.7	16	19.0	-
**Know manifestations of SCD**	-	-	-	-	0.012
No	42	47.0	21	25.0	-
Partially	24	27.0	31	37.0	-
Yes	23	26.0	31	37.0	-
**Know complications of SCD**	-	-	-	-	0.032
No	70	79.0	53	64.0	-
Yes	19	21.0	30	36.0	-
**Know what EPI is**	-	-	-	-	0.048
No	69	77.5	50	60.0	-
Yes	14	15.7	22	27.0	-
Vaguely	6	6.7	11	13.0	-
**What was the most annoying during immunisation session?**	-	-	-	-	0.084
Very long waiting time	43	53.8	46	61.0	-
Distance to vaccination centres	20	25.0	11	15.0	-
Information access	14	17.5	9	12.0	-
Bad reception	3	3.8	9	12.0	-

SCD, sickle cell disease; EPI, Expanded Programme of Immunisation.

* , Independent Chi-square test; Student’s *t*-test; Fisher’s exact test.

† , Median(ET).

Nevertheless, the difference observed was not statistically significant (*p* = 0.433).

#### Clinical data and immunisation status

There was no association between the type of recurrent infection and the immunisation status of patients (*p* = 0.641), nor was there any association with the PPSV23 immunisation status in children with SCD. Nevertheless, we found that there was a significant association between the type of infection and the immunisation status regarding the Penta booster dose (*p* = 0.038).

There was no association between socioeconomic status and age of discovery of the sickle cell status, HbS level or number of transfusions. No association with EPI schedule, Penta, or PPSV23 coverage was shown. However, data analysis on knowledge of complications revealed that knowledge of complications was different when comparing families by socioeconomic status. Thus, we observed that 35.0% of median-income parents or legal guardians exhibited awareness about complications of SCD. That rate was 21.3% for low-income parents, while it was as high as 66.7% for high-income parents (*p* = 0.048).

Knowledge of the EPI schedule was different when comparing parents on the basis of their socioeconomic status. Knowledge of the EPI schedule was 15.0% among low-income participants. It was 23.8% among median-income parents and 100.0% for parents from the high-income category. We found that knowledge about immunisation came mainly from medical personnel regardless of the socioeconomic status of parents. In other words, there was no significant difference among families with regard to knowledge about immunisation (*p* = 0.755).

Regarding rumours about immunisation, there was no difference between socioeconomic groups, as all groups of parents had already heard rumours about immunisation (*p* = 0.899). Interestingly, 18.2% of low-income participants, 8.1% of median-income participants, and 50.0% of high-income participants expressed agreement with the rumours.

We saw no significant difference between healthcare facilities visited by different social groups (*p* = 0.444). Likewise, we saw no difference across the different socioeconomic categories in their judgement of how immunisation sessions were organised in healthcare facilities.

Our study also revealed that rumours had no influence on the PPSV23 vaccine status (*p* = 0.669) and no association was found between polio campaigns and rumours.

#### Impact of factors on immunisation rates

Children for whom parents claimed to carry out the immunisation in private medical facilities had better immunisation coverage against PPSV23 (54.5%) compared to children for whom parents said that they had them vaccinated in public health facilities (25.0%) (*p* = 0.046).

Our study shows a significant association between the EPI status of children and the type of nearest immunisation centre (Table 6). Based on EPI schedule, the analysis of immunisation status for the surveyed sample of SCD children revealed that children living close to a public immunisation centre were better immunised (91.4%) compared to those living close to other types of public health (50.0%) or private health facilities (70.0%) with *p* = 0.008. There was no association between the type of immunisation centre and the Penta booster (*p* = 0.67), nor with PPSV23 (*p* = 0.408).

**TABLE 6:** Comparison between complete of vaccination, out of expanded programme of immunisation schedule, and other variables, in children with sickle cell disease living in Libreville.

Characteristics	Complete vaccination out of EPI				*P* *
	No (*n* = 35)†		Yes (*n* = 72)†		
	*n*	%	*n*	%	
**Sanitary facilities**	-	-	-	-	0.004
Public health centres	32	91.4	47	65.0	-
Private clinic	3	8.6	25	35.0	-
**Immunisation status according to EPI**	-	-	-	-	< 0.001
Good	25	71.0	70	97.2	-
Bad	10	29.0	2	2.8	-
**Usual Haemoglobin level low than 7 g/mm^3^**	-	-	-	-	0.014
No	13	37.1	57	79.2	-
Yes	5	14.3	3	4.2	-
Unknown	17	48.6	12	16.7	-
**What was the most annoying during immunisation session?**	-	-	-	-	0.013
Very long waiting time	25	71.4	33	45.8	-
Distance to vaccination centres	2	5.7	13	18.0	-
Information access	1	2.9	14	19.4	-
Bad reception	2	5.7	6	8.3	-
No opinion	5	14.3	6	8.3	-

EPI, Expanded Programme of Immunisation.

* , Independent Chi-square test; Student’s *t*-test; Fisher’s exact test.

† , Median(ET).

Overall, 12.0% of parents had knowledge about the complications of SCD. Furthermore, we found an association between the frequency of complications and the knowledge of parents about complications. The lack of knowledge about complications was more common among parents for whom children had not yet experienced health complications 112 (65.1%). Children with sickle cell disease who had complications were found to have parents with better knowledge about SCD complications, totalling 11 (6.4%) in our study, with *p* = 0.032.

### Attitude of parents

To the question ‘do you think this immunisation session was well organised?’ 114 (66.8%, [59.2–73.3]) responded ‘Yes’, 26 (15.1%, [09.8–20.5]) said ‘No’, and 32 (18.6%, [18.8–24.4]) replied ‘I don’t know’. We further found that the worst moments parents spent in public facilities were at the reception desk and the extremely long waiting time (Table 2).

In our final model, we found that Penta booster and the knowledge of parents about SCD complications were associated with complete immunisation in children (Table 7).

**TABLE 7:** Factors associated with immunisation coverage in children with SCD in Libreville, Gabon.

Variable	Initial model			Final model		
	OR	CI_95%_	*p*	OR	CI_95%_	*p*
Marital status of parents: Unmarried	0.1116	0.01–1.15	0.88847	-	-	-
Marital status of parents: Married	1.0515	0.50–2482.00	0.13318	-	-	-
Socioeconomic status: High income	0.8471	0.20–26.00	0.64122	-	-	-
Socioeconomic status: Middle income	0.4930	0.20–5100.00	0.41730	-	-	-
Type of healthcare facility: Public	0.1164	0.01–4.00	0.84732	-	-	-
Booster Penta: Yes	1.5099	0.70–9.90	0.07220	5.047	1.0–24.6	0.045
Knowledge of SCD complications: Yes	1.0703	2.10–98.00	0.06696	3.028	1.0–08.8	0.043

SCD, sickle cell disease; CI, confidence interval; OR, odds ratio.

## Discussion

The objective of this study was fourfold: (1) determine the immunisation coverage of children with SCD regularly seen by paediatricians in Libreville, (2) investigate the determinants of this coverage through knowledge of parents, (3) unravel the attitudes of parents or legal guardians towards their children’s health condition, and (4) the means to prevent infectious diseases and complications in SCD children. Our study could have some limitations. The first limitation may be that by selecting children in a given paediatric ward, we did not access data from SCD children who were not regularly screened for complications. The second limitation may be that some parents found the questionnaire and the interview too long, even though we did not exceed 20 minutes. Some aspects, such as social representations or the influence of the age of the mother on the immunisation status of children, could have been considered in this study, but we did not. Also, the fact that mothers are often the ones accompanying children to immunisation sessions makes very much sense to include that factor in our study. However, this factor was not looked into.

### Immunisation coverage

In our study, based on the national EPI schedule, the immunisation status was considered good for 94 SCD children out of 107, which represented 87.9% (95% CI = 79.8–93.1). This rate of immunisation coverage in children with SCD was at least twice better than the national immunisation coverage, which averaged around 32% at the time of this study. A similar observation has been made in other African countries, including Cameroon, where immunisation coverage was 96.0%^3^, Burkina Faso, 97.5%^22,23^ coverage, and DRC, 88.5%^3^ coverage. That observation clearly shows that parents of children with SCD are very much aware of the risk of infections to which their fragile and precious children could be exposed and are taking appropriate steps to tackle the issue. Parents or legal guardians of children with SCD understandably worry more about the health of their children compared to others. Probably, SCD crises and complications are so worrisome that parents or legal guardians are ready to do whatever it takes to prevent infections and, therefore, limit the risks of complications.

The immunisation status of children with SCD was neither associated with the type of facility (private or public) visited by patients nor was it with the social status of parents. Nonetheless, we showed that it was associated with the marital status of parents, leading to the conclusion that the monitoring of children’s immunisation schedule is better performed or most likely to be performed when the two parents are living together. As observed in other studies, ignorance and the prohibitive cost of vaccines^24,25,26,27^ were the two major obstacles to completing supplemental immunisation in addition to those existing on the regular immunisation schedule of EPI.

### Knowledge of the disease and immunisation

The low level of knowledge of parents or legal guardians about the disease and immunisation has been highlighted in this study. Knowledge of the disease and immunisation differed tremendously according to the socioeconomic status of parents or legal guardians of children with SCD. Parents who were aware of SCD complications were those whose children had developed complications at some point in the medical history of their child. This observation speaks to the urgent need and importance of educating parents on improving their knowledge about the disease and ways to avoid complications in children. Thus, governments need to implement a therapeutic education system for parents with SCD children along with patients themselves because this has already proven to be effective elsewhere.^28,29^

### Immunisation access

The main complaint of parents was the quality of reception in public immunisation facilities, which led parents to take their children to private healthcare facilities with, unfortunately, high price tags for services. It is, therefore, necessary for the government to train healthcare staff to improve reception quality in public health facilities in Gabon. Such training could include professional development courses or workshops on topics such as hospitality, empathy, and communication skills. In the United States (US), out of 266 children with SCD, 41% received influenza immunisation. In this study, only 5.3% of those children were up-to-date with all preventive care, which included immunisation against influenza, use of prophylactic antibiotics, and administration of trans-cranial Doppler ultrasound.^30^ In Gabon, immunisation is free only during the first year of life. After this period and because of the prohibitive cost of vaccines, access to immunisation becomes a challenge for Gabonese families, including for families with SCD children. This situation exposed SCD children to major health complications and risks. With the development of the National Health Insurance and Social Guarantee Fund of Gabon, which take into account the care of SCD patients, we can expect the cost of immunisation to be reduced in future years.

### Limitation

This study has some limitations. The context of recruitment was different for each patient; the context of recruitment varied for each patient; in some cases, it might involve a routine visit, an acute crisis, or while hospitalised, depending on the reason. Patients were only recruited in some Libreville healthcare centres or clinics, but many SCD children are living in other cities or rural places in Gabon. These results are, therefore, not completely representative of all Gabonese children living with SCD. Therefore, we believe it makes an important contribution to evaluating immunisation coverage of SCD children and its determinants.

## Conclusion

According to EPI schedule, immunisation coverage of children with SCD was better than the national immunisation coverage in Gabon. The marital status of parents, the lack of knowledge, and the prohibitive cost of vaccines were predictor factors that could affect the level of immunisation coverage of children with SCD. It seems apparent that immunisation programmes in public health facilities must be improved. Finally, the contribution of health insurance may enhance immunisation of SCD children in Gabon.
